# Identification of two distinct genes at the vertebrate *TRPC2 *locus and their characterisation in a marsupial and a monotreme

**DOI:** 10.1186/1471-2199-12-39

**Published:** 2011-08-19

**Authors:** Stephen Frankenberg, Nanette Y Schneider, Terrence P Fletcher, Geoffrey Shaw, Marilyn B Renfree

**Affiliations:** 1 Department of Zoology, The University of Melbourne, Melbourne, Victoria, 3010, Australia

## Abstract

**Background:**

The vomeronasal organ (VNO) detects pheromones via two large families of vomeronasal receptors: vomeronasal receptor 1 (V1R) and vomeronasal receptor 2 (V2R). Both VRs have a common receptor activation cascade involving transient receptor potential channel, subfamily C, member 2 (TRPC2).

**Results:**

We characterised the *TRPC2 *locus in a marsupial, the tammar wallaby (*Macropus eugenii*), and identified two independently regulated genes not previously recognised as distinct. 3'-located exons comprise bona fide *TRPC2 *whilst 5'-located exons, previously identified as part of *TRPC2*, comprise a distinct gene, which we term *XNDR *(*X*RCC1 *N*-terminal *d*omain-*r*elated). The two genes show contrasting expression patterns in the tammar: *TRPC2 *is specifically expressed in adult and developing VNO, whereas *XNDR *is widely expressed in many tissues suggesting a non-VNO-specific role. Strong expression of *TRPC2 *was detected only after about day 30 post-partum, suggesting that the VNO may not be functional during early pouch life of the tammar. Similarly restricted expression of *TRPC2 *and widespread expression of *XNDR *was also detected in the platypus. Bioinformatic analysis of the genomes of a wide range of species suggests that the identity of *XNDR *and *TRPC2 *as distinct genes is conserved among vertebrates. Finally, we analysed the promoter of mammalian *TRPC2 *and identified a conserved binding site for NHLH1, a transcription factor previously implicated in VNO receptor neuron development.

**Conclusions:**

Two functionally distinct vertebrate genes-*XNDR *and *TRPC2 *- occupy a genomic locus that was previously defined as a single gene in the mouse. The former is widely expressed with a putative role in DNA repair, while the latter shows VNO-specific expression under the probable regulation of NHLH1.

## Background

The vomeronasal organ (VNO) is a paired tubular organ that is found in the nasal cavity of most tetrapods [1,2]. It lies in the tissue on either side of the nasal septum, stretching along its sides towards the back of the nasal cavity. The VNO is thought mainly to detect pheromones or pheromone blends [3], but it also detects some environmental odors [4,5]. Functioning of the VNO requires functional receptor cells with connections to the brain and all parts of the receptor activation cascade. Two families of vomeronasal receptors (VRs)-V1Rs and V2Rs-are specific to the VNO and the number of family members varies greatly between species (e.g. [6-10], making comparative studies difficult. Not all mammals have intact receptors of both families. The dog (*Canis familiaris*) and the cow (*Bos taurus*), for example, only have intact *V1R *genes and lack functional *V2R *genes [8,9]. Both vomeronasal receptors are thought to use a common receptor activation cascade that depends on the transient receptor potential channel protein, subfamily C, member 2 (TRPC2) [11,12]. TRPC2 is one of seven known TRPCs [13]. While the activation process is still not fully understood, the TRPC2 channel is thought to be modulated through phospholipase C [14]. *TRPC2 *represents its own gene subfamily as it is quite dissimilar to the other *TRPC*s in sequence and function [15]. To date, full-length transcripts of *TRPC2 *have been described for the mouse (*Mus musculus*) [16], rat (*Rattus norvegicus*) [12], New World monkeys [17-19], cow [20], Californian sea lion (*Zalophus californianus*) [21] and zebrafish (*Danio rerio*) [22], whereas *TRPC2 *in human (*Homo sapiens*) [23], Old World monkeys [17,19], dolphin (*Tursiops truncatus*), little brown bat (*Myotis lucifugus*), flying fox (*Pteropus vampyrus*) [10], fin whale (*Balaenoptera physalus*), harbour seal (*Phoca vitulina*) and river otter (*Lutra lutra*) [21] contains stop codons, indicating a disabled pseudogene. Partial sequences of *TRPC2 *have been characterised for the elephant shark (*Callorhinchus milii*) and sea lamprey (*Petromyzon marinus*) but not in non-vertebrate chordates such as lancelet (*Branchiostoma floridae*) and sea squirt (*Ciona intestinalis*), suggesting that *TRPC2 *was already present in the olfactory epithelium of the common ancestor of all vertebrates [24].

In the rat VNO, Trpc2 is prominently and selectively localised to the microvilli of the receptor cells, which are thought to be the binding site of pheromones [12,25]. *Trpc2 *is expressed strongly in the VNO and in only a very small population of cells within the main olfactory epithelium (MOE) [12]. Mouse Trpc2 is localised to the anterior part of the sperm head where it is thought to be involved in the acrosome reaction, as Ca^2+ ^entry and the acrosome reaction are both blocked by an antibody to Trpc2 [26]. However, *Trpc2*^-/- ^null mutant mice are fertile, so the importance of Trpc2 for the acrosome reaction is in doubt [11,27]. *Trpc2*^-/- ^mice do, however, provide dramatic evidence for the importance of Trpc2 in VNO function [11,27]. *Trpc2*^-/- ^male mice fail to initiate attack behavior at the approach of an intruding male and fail to establish a rank order [11,27]. *Trpc2*^-/- ^males also mount other males far more than normal males do. *TRPC2*^-/- ^null mutant mice show not only a change in behaviour [11,27], but also exhibit differential hypotrophy of the glomerular layer of the AOB, with the anterior portion of the glomerular layer (innervated by V1R cells) resembling that of wild-type mice, and the posterior portion (innervated by V2R cells) reduced or absent [28].

Vannier et al. 1999 [16] identified two transcripts of *mTrpc2 *(*mTRPC2A *and *mTRPC2B*) that were exclusively expressed in testis, whereas a later study [29] failed to find these two transcripts but described two shorter transcripts called *mTRPC2α *and *mTRPC2β*. The predicted proteins encoded by *mTRPC2α *and *mTRPC2β *lacked the N-termini of mTRPC2A and mTRPC2B and resembled more rat Trpc2 and other TRPC proteins. *mTRPC2β *was found to be strongly expressed in the VNO and only weakly expressed in testis, suggesting it may represent the more biologically relevant variant.

As *TRPC2 *is relatively conserved among species (e.g. [24]) in contrast to *V1R *or *V2R *genes (e.g. [6-10]), it is more suited for investigating the evolution of the VNR pathway in vertebrates and especially in mammals. To date, *TRPC2 *has only been studied in eutherian mammals but not in marsupials or monotremes. Further data may reveal whether a longer transcript also exists in species other than mouse or whether the shorter transcript mTRPC2β is more similar to other species as described in rat. Using genome sequence data from two divergent marsupials, the tammar wallaby (*Macropus eugenii*) and the gray short-tailed opossum (*Monodelphis domestica*), we analysed the *TRPC2 *locus of marsupials and compared it to that of other vertebrates. We also include an analysis of this locus in another divergent mammal, the platypus (*Ornithorhyncus anatinus*). By a combination of cDNA cloning, expression analysis and bioinformatics, we show that the locus orthologous to mouse *Trpc2 *is comprised of two distinct genes that are regulated independently.

## Results

### The *TRPC2 *locus is comprised of two independently regulated genes

**Figure 1:**
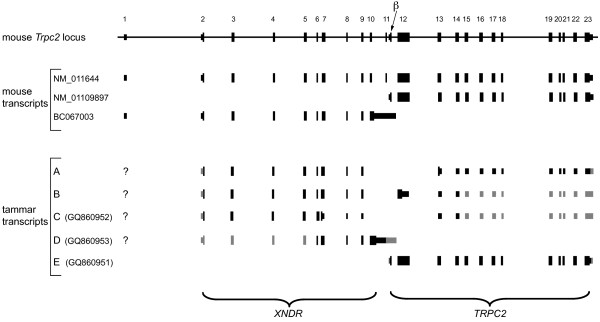
**Exons and splice variants of tammar *TRPC2 *aligned with exons at the murine *Trpc2 *locus**. All exons and intergenic distances of the murine *Trpc2 *locus are shown approximately to scale. The exon structure of three murine transcripts and their GenBank accession numbers are shown. Exons are numbered according to [GenBank:NM_011644], except Exon β, which is the first exon of [GenBank:NM_01109897]. Putative tammar transcripts (A, B, C, D & E) are also shown, with exons aligned to their murine orthologues. Tammar exons shown in black represent splicing structures confirmed by RT-PCR, while those in grey are predicted. Transcripts C and D represent isoforms of tammar *XNDR *([Genbank:GQ860952] and [GenBank:GQ860953], respectively), while transcript E represents tammar *TRPC2 *([GenBank:GQ860951]). The coding exons corresponding to *XNDR *and *TRPC2 *are indicated by parentheses (bottom).

In order to investigate the presence of *TRPC2 *orthologues in marsupials, we performed BLAST searches of genomic databases of the grey short-tailed opossum and the tammar. The longest murine *Trpc2 *transcript [GenBank:NM_011644] contains 23 exons, with the coding sequence spanning Exons 2-23 (Figure 1). Sequences orthologous to all murine coding exons were identified in the genomes of both tammar and opossum. For both species, splice donor and acceptors sites were identified for all coding exons with the exception of the 3' donor site of Exon 10 and the 5' acceptor site of Exon 11. Retention of the intron between Exons 10 and 11 was predicted to result in a truncated protein. Nevertheless, Exons 12-23 of both marsupial species had an uninterrupted open reading frame (ORF).

To elucidate the splicing arrangement of *TRPC2 *transcripts in the tammar, RT-PCR was performed on adult female VNO-derived cDNA. RT-PCR using primers specific to Exons 2 (1F) and 23 (1R) produced two faint bands of slightly differing size (not shown). This PCR was used as a template for several nested PCRs to amplify fragments of *TRPC2 *cDNA. Using nested primers specific to Exons 2 (2F) and 23 (2R), one fragment was cloned that was sequenced and found to represent Exons 2-9 and 13-23 in contiguity (see predicted tammar transcript A (Figure 1)). PCR using nested primers specific to Exons 2 (2F) and 14 (5R) yielded two more cloned products of differing size. Sequencing of the first of these showed it to represent Exons 2-9 and 12-14 in contiguity (see predicted tammar transcript B (Figure 1)). Sequencing of the second clone showed it to be similar to the first except that Exon 6 was longer (resulting from the use of an alternate splice donor site) and that Exon 12 was omitted (see predicted tammar transcript C (Figure 1)).

The predicted ORFs of all three of the above clones would result in substantially truncated proteins. Because we were unable to amplify any products containing putative Exons 10 and/or 11, an additional RT-PCR was performed using primers specific to Exon 6 (3F) and the 3' part of Exon 11 (6R). This resulted in a cloned fragment representing Exons 6-11 but including intronic sequence between Exons 10 and 11 (see predicted tammar transcript D (Figure 1)). Sequence from this clone also encoded a predicted truncated protein. From our RT-PCR results and the apparent absence of appropriate splice sites, we conclude that in marsupials no intron is ever spliced out between the regions orthologous to murine Exons 10 and 11.

**Figure 2:**
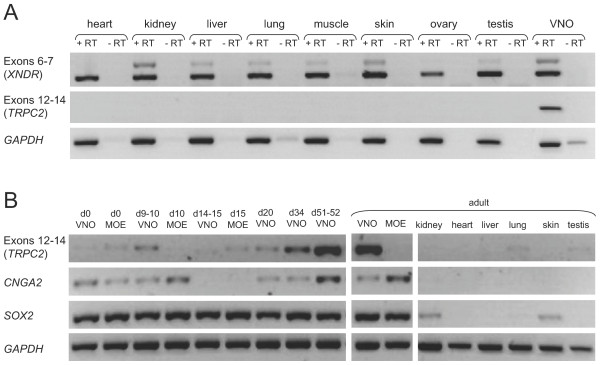
**Expression of exons at the tammar *TRPC2 *locus in adult and pouch young**. **A**. Exons 6-7 (primers 3F & 4R) were expressed in a range of adult tammar tissues whereas expression of Exons 12-14 (primers 4F & 5R) was specific to the VNO. The two bands for Exons 6-7 resulted from the two alternate splice donor sites of Exon 6. "+ RT" and "- RT" denote inclusion and omission of reverse transcriptase, respectively. **B**. Expression of Exons 12-14 in the VNO was detected weakly in VNO and main olfactory epithelium (MOE) samples at day 0, day 10, day 15 and day 20 postpartum and more strongly from day 34 postpartum. In the adult, expression was highly specific to the VNO, absent in MOE, and only very weakly detected in lung and testis.

The conservation of predicted amino acid sequence from both Exons 2-9 and 12-23 suggested that both regions are functional but do not encode the same protein. Evidence from previous reports [12,29] suggested that a distinct promoter downstream of Exons 2-9 drives expression of Exons 12-23 specifically in the VNO. In fact, in no species other than the mouse could we find any evidence for a transcript with a continuous ORF that included both regions. We therefore compared expression by non-nested RT-PCR using primer pairs specific to each region. Primers specific to Exons 6 (3F) and 7 (4R) yielded two products (resulting from each of the alternate splice donor sites of Exon 6 (above)) from a broad range of adult tissues, whereas primers specific to Exons 12 (4F) and 14 (5R) yielded a product only from adult VNO (Figure 2A).

We next examined the onset of VNO-specific expression (Exons 12-14) in early tammar pouch young. Because of the difficulty in separating VNO from main olfactory epithelium (MOE) and other tissues in the nasal region of the smallest pouch young (the neonate weighs only 400 mg), we also tested markers of sensory neurons (*SOX2 *[30]) and MOE (*CNGA2 *(also called *oCNC1*) [31]). RT-PCR product for Exons 12-14 was detected strongly in the VNO of day 34 and day 52 pouch young, very weakly in some earlier pouch young VNO and MOE samples and very weakly in adult liver and lung (Figure 2B). Detection of *CNGA2 *in both VNO and MOE samples of some early pouch young suggested that there may have been cross-contamination of these tissues or that VNO- and MOE-specific receptor neurons have not fully differentiated from each other at these very early stages of development.

**Figure 3:**
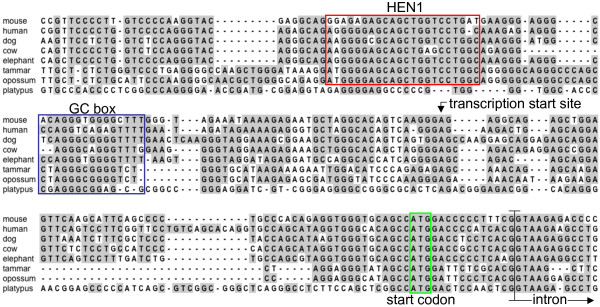
**Alignment of the putative promoter and Exon β of *TRPC2 *from selected mammal species**. High sequence conservation is observed near the boundary between Exon β and the adjacent intron, as well as at a putative HEN1 binding site and GC box. The indicated transcription start site corresponds to that of the mouse *Trpc2β *transcript.

Our results indicated that a second, VNO-specific promoter lies upstream of tammar Exon 12, as previously postulated for the mouse [32]. We found that the putative first exon-"Exon β"-of a mouse VNO-specific transcript [32] is also conserved in sequence upstream of Exon 12 in the tammar wallaby as well as many other mammal species (Figure 3). RT-PCR of tammar VNO-derived cDNA using primers specific to Exons β (5F) and 23 (3R) revealed a full-length open reading frame, represented by transcript E (Figure 1) [GenBank:GQ860951].

The essential domains for TRPC2 function, including the transmembrane and cytoplasmic domains, are encoded by Exons β-23. Exons 2-9 were predicted to encode a protein with similarities to the N-terminal domain of XRCC1 (X-ray repair complementing defective repair in Chinese hamster cells 1). Our expression and bioinformatic data indicate that the latter exons comprise a gene distinct from *TRPC2*, which we term *XNDR *(Xrcc1 N-terminal domain related). Murine *Xndr *is therefore represented by murine transcript [GenBank:BC067003] (Figure 1). Hence we refer to transcripts comprised of Exons β-23 as representing *TRPC2 *(corresponding to murine transcript [GenBank:NM_001109897] (Figure 1)) and transcripts encompassing both regions as representing *XNDR-TRPC2 *(corresponding to murine transcript [GenBank:NM_011644] (Figure 1)). In the mouse, Xndr-Trpc2 (previously called isoform 1 of Trpc2) is thus encoded by transcripts initiated by the *Xndr *promoter in which splicing occurs between the donor site of Exon 10 and the acceptor site of Exon 11, whereas these splice sites are not used in transcripts that encode Xndr.

### *XNDR *and *TRPC2 *are both highly conserved among vertebrates

**Figure 4:**
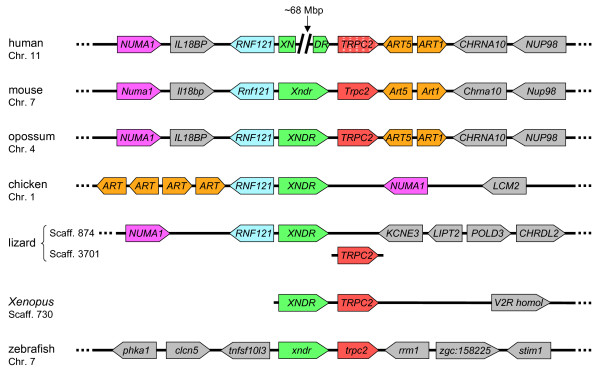
**Comparison of gene synteny in orthologous regions from various vertebrate species**. *TRPC2 *flanks *XNDR *in all species shown except chicken, in which it is apparently absent, and lizard, in which it occupies a different locus. In human, *XNDR *is disrupted by approximately 68 Mb of intervening sequence, while *TRPC2 *is a disabled pseudogene. (Gene sizes and intergenic distances are not shown to scale.).

We examined the evolution of the XNDR/TRPC2 locus by comparing the assembled genomes of a broad range of vertebrate species available on the UCSC browser. Orthologues of *XNDR *appear to be present in all jawed vertebrates ranging from teleost fishes to mammals (Figure 4). However, we were unable to detect orthologues of *XNDR *in lower deuterostomes such as lancelet and sea squirt, in which the closest homologues appear to represent orthologues of *XRCC1 *(data not shown). In human and chimpanzee (*Pan troglodytes*), the gene is disrupted, with Exons 1-5 located approximately 68 Mbp distant to Exons 6-10 on chromosome 11 of both species. This is a relatively recent event, however, as all 10 exons are normally arranged in another hominid, the orangutan, as well as in other primates, with no disruption of the ORF (not shown). The N-terminal region of XNDR encoded by Exons 2-5 is the more highly conserved (data not shown) and presumably the more functionally important. Thus the non-disrupted ORF of Exons 2-5 may still be functional in both human and chimpanzee.

*TRPC2 *orthologues in human and other catarrhine primates have a disrupted ORF, as previously reported [17,19,23]. In the chimpanzee, Exons 12-19 appear to have been completely lost, while in the orangutan (*Pongo pygmaeus*), a region corresponding to ~4.75 kb in human and containing Exons 16-18 is deleted. In the Rhesus macaque (*Macaca mulatta*), all exons are present but Exon 16 is inverted (data not shown).

*XNDR *and *TRPC2 *flank each other in all species in which the relative positions of both genes could be determined, with the exception of the anole lizard (*Anolis carolinensis*) in which they occupy separate genomic scaffolds (Figure 4). The close proximity of *XNDR *and *TRPC2 *appears to be ancestral, as it is also found in teleost fishes (Figure 4). There is also a second homologue of *TRPC2 *[GenBank:XM_683311] located on chromosome 15 of the zebrafish genome that does not appear to be present in other teleost species.

### *XNDR *and *TRPC2 *are differentially regulated in the platypus

**Figure 5:**
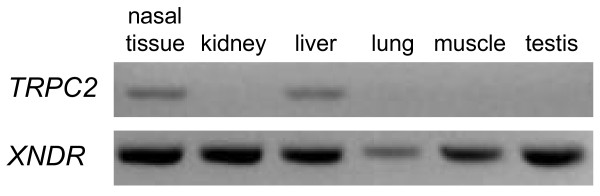
**Expression of *TRPC2 *and *XNDR *in the platypus**. Platypus *TRPC2 *expression was detected in a sample of nasal tissue and in liver, but not in any other adult tissues. By contrast, *XNDR *expression was strongly detected in all adult tissues tested.

To confirm that independent transcriptional regulation of *TRPC2 *and *XNDR *is conserved at least among mammals, we examined their expression in the platypus, one of the five living species of the third extant sub-order of mammals. Sequences orthologous to Exons 2-10, representing the complete coding sequence of *XNDR*, were identified in the platypus genome, along with Exons β, Exons 13-14, Exons 17-20 and part of Exon 23 of *TRPC2 *(data not shown). Although the platypus genome build is incomplete in this region, continuous sequence separates Exons 10 and β, showing that *TRPC2 *and *XNDR *flank each other. Forward primers designed within Exons 9 and β and reverse primers designed within Exons 10 and 13 (Table 1) were used to test expression in adult tissues, including nasal tissue believed to contain the VNO, by RT-PCR. Platypus *XNDR *(Exons 9-10; 440-bp product) was detected in all tissues tested, whereas *TRPC2 *(Exons β-13; 881-bp product) was only detected in nasal tissue and liver (Figure 5). The sequence of the latter gel-extracted product has been deposited in GenBank [GenBank:HQ113234]. RT-PCR using the Exon 9 forward primer and the Exon 13 reverse primer did not yield any detectable product (not shown), suggesting that expression of an *XNDR-TRPC2 *transcript is low or absent in the platypus.

**Table 1:** Sequences of tammar-specific primers

gene	name	sequence	location of primer binding
*XNDR-*	1F	GGCTTATGGCTGCTCTCTTTCTGTGAC	Exon 2
*TRPC2*	2F	TCCTCATGGCTCCTGTGAAGATCAG	Exon 2
	3F	CGGAGAACATTCTTCCCAGA	Exon 6
	4F	TTCCCTCTCCCGAATTAACA	Exon 12
	5F	AGCAAACAGAGCAAGACTAGGAGGGTATAG	Exon β
	1R	TTCTTCCTGAAACCCCCTGTGAATC	Exon 23
	2R	TGAATCTCCACTTGGGCAGCGG	Exon 23
	3R	ATAGGTGGCACGGGGTTAGGTCAG	Exon 23
	4R	ACATCTGAGCTGGCTGTGTG	Exon 7
	5R	GCCAAGTTTCCACACCAGAT	Exon 14
	6R	TTGGGTTGGGGCTGGAATAGTAATG	Exon 11

*CNGA2*	for	TGTGCTTGATCCTGCTGGAGACTG	
	rev	ACCACATCAGTGGGGATGACGG	

*SOX2*	for	TGGAGCAACGGCGGCTACGG	
	rev	CCTGGAGTGCGACGACGAGG	

*GAPDH*	for	CCTACTCCCAATGTATCTGTTGTGG	
	rev	GGTGGAACTCCTTTTTTGACTGG	

### The therian *TRPC2 *promoter contains a putative NHLH1 transcription factor binding site

To characterise the promoter that may be responsible for specific expression of *TRPC2 *in the vomeronasal organ, we compared genomic sequence immediately upstream of the first exon (β) of *TRPC2 *from a range of vertebrate species. When we compared those of mammals, two sites were identified as likely candidates: a HEN1 site and a GC box (Figure 3). The former shows strong similarity to the consensus binding site for NHLH1 (also called HEN1 or NSCL1), a neural-specific basic helix-loop-helix (bHLH) transcription factor [33]. The HEN1 site was well conserved among eutherians and marsupials, but not in the platypus (Figure 3) or in other vertebrates (not shown).

## Discussion

*Trpc2 *was originally identified as encoding a VNO neuron-specific ion channel in the rat [12]. This transcript [GenBank:AF136401] corresponds to the transcript that we define here as *TRPC2 *proper. Two subsequent reports, however, identified additional variants of murine "*Trpc2*" transcript variants that were termed *Trpc2A*, *Trpc2B *[16], *Trcpc2α *and *Trpc2β *[29]. *Trpc2β *was isolated by RACE-PCR from vomeronasal tissue and corresponds to the original rat *Trpc2 *transcript. Each of the other transcripts included exons from *Xndr *and, notably, was amplified from non-VNO-derived cDNA. The primers and methods used would have specifically favoured amplification of cDNA fragments of *Xndr-Trpc2*, rather than *Xndr *or *Trpc2*, even if they were at low abundance. Although Hofmann et al [29] reported that only *Trpc2β *was specifically expressed in the VNO and that two independent promoters are likely to be functional, until now there has been no formal recognition that *Xndr-Trpc2 *represents a fusion of two distinct genes and may be biologically irrelevant. At the time of preparing this manuscript, exons from both genes are still presented as defining "*Trpc2*" by the Entrez Gene database on the NCBI website.

In this study, we have presented several pieces of evidence to support the recognition of two distinct genes-*XNDR *and *TRPC2 *- at this locus in mammals. First, splice sites necessary for the full-length ORF of mouse *Xndr-Trpc2 *are not conserved in most other species, rendering it implausible that *XNDR-TRPC2 *has any conserved role among vertebrates. Second, comparisons by RT-PCR using primers specific to either *XNDR *or *TRPC2 *in the tammar wallaby and the platypus clearly supported the original evidence [29] that two independent promoters respectively regulate widespread (*XNDR*) and VNO-specific (*TRPC2*) expression. Third, analysis of a range of vertebrate genomes shows that the two genes have evolved independently. In the anole lizard, *XNDR *and *TRPC2 *are both conserved but occupy separate genomic scaffolds. In the chicken, *XNDR *is conserved whereas *TRPC2 *appears to be lost, or at least does not flank *XNDR*. In catarrhine primates, at least the first 4 coding exons of *XNDR *are conserved and presumably functional, despite the loss of functionality of *TRPC2*. Together, our data provide overwhelming evidence for identifying *XNDR *as a novel gene distinct from *TRPC2*. *XNDR *encodes a predicted protein with similarities to the N-terminal domain of XRCC1 (X-ray repair complementing defective repair in Chinese hamster cells 1). XRCC1 has a key role in DNA base excision repair (reviewed [34]); thus it is possible XNDR has a similar role.

*TRPC2 *expression was highly specific to the VNO in the tammar, being highly expressed not only in the adult, but also in pouch young as early as day 34 *post partum*. Expression was only weakly detected at earlier stages, possibly due to the small proportion of expressing cells. Marsupials deliver highly altricial young that are equivalent to the eutherian fetus and complete most of their development *post partum *[35]. The surrounding tissue that was also dissected along with the VNO at these earlier stages may have diluted the proportion of *TRPC2 *transcripts in our samples. At birth, Goα protein was confined to only a limited number of cells [36]. It thus seems likely that the VNO does not begin to function until around day 30 *post partum *in the tammar. This would make the main olfactory system the primary candidate for perception of pouch odours that guide the neonatal tammar into the pouch at birth [36]. Further studies will be needed to confirm whether the VNO is functional earlier in development in these highly altricial young.

We identified NHLH1 as a candidate regulator of *TRPC2 *expression. Mouse *Nhlh1 *is expressed specifically in the developing nervous system, peaking around the period of E10 to 12.5, and marks early post-mitotic neuroblasts [37,38]. Significantly, expression of *NHLH1 *becomes restricted during later stages to olfactory and vomeronasal receptor neurons [39]. Mammalian NHLH1 and NHLH2 are encoded by paralogous genes and are almost identical in their C-terminal bHLH domain but highly divergent in their N-terminal domain [40]. Murine *Nhlh2 *is also expressed in the developing nervous system, peaking at around E11.5 [40]. In the NCBI UniGene gene expression profile estimated by expressed sequence tag (EST) frequency in adult tissues, expression of *Nhlh1*, but not *Nhlh2*, is highly restricted to the nasopharynx http://www.ncbi.nlm.nih.gov/UniGene/ESTProfileViewer.cgi?uglist=Mm.2474, consistent with a specific role for NHLH1 in mammalian olfactory and vomeronasal receptor neurons. We therefore propose that NHLH1 specifically regulates *TRPC2 *expression in VNO receptor neurons in mammals. We also found conserved E-box consensus sequences in the *TRPC2 *promoter of some teleost fishes (not shown), suggesting that other bHLH transcription factors may be involved in regulating *TRPC2 *in other species. The genomes of teleost fishes only appear to contain an orthologue of *NHLH2 *but not *NHLH1*, suggesting that *NHLH2 *represents the ancestral gene. Both jawed and jawless fishes only have a single olfactory organ, but the genetic components of a vomeronasal sensory system are nevertheless present in both groups [24]. It is possible that the evolution of *NHLH1 *is linked to the evolution of a morphologically more complex olfactory system in the tetrapod lineage.

## Conclusions

By a combination of expression analysis, genomic analysis and a critical assessment of previous literature, our study clearly demonstrates that the locus formerly defined as encompassing a single gene in reality comprises two distinct genes: *XNDR *and *TRPC2*. This distinction is important for future studies, especially for those comparing VNO function among divergent vertebrate species. *XNDR *is broadly expressed and has a possible role in DNA repair, while *TRPC2 *is specifically expressed in the VNO under the probable regulation of NHLH1. The expression profile of *TRPC2 *in the tammar wallaby suggests that there is no *TRPC2*-dependent role for the VNO during early pouch life.

## Methods

### Animals

Tammar wallabies were obtained from our breeding colony held under permits from the Victorian Department of Natural Resources and Environment. Platypus tissues were collected from two adult males trapped in the Murrumbidgee River, NSW, under a permit from NSW National Parks & Wildlife Service. Adult animals (tammar and platypus) were euthenised by an overdose of sodium pentobarbitone. Tammar pouch young younger than 40 days (< 1 g) (that are heterothermic [35]) were cooled then decapitated. All experiments were approved by the University of Melbourne Animal Experimentation Ethics Committees and all animal handling and husbandry were in accordance with the National Health and Medical Research Council of Australia (2004) guidelines.

### Tissues

Heart, kidney, liver, lung, muscle, skin, ovary, testis, retina, tongue (from the tip), MOE and VNO tissue were collected from adult female tammar wallabies. VNO tissue was collected from pouch young of age 0, 10, 15, 20, 34 and 52 days *post partum *(each n = 1). MOE was collected from pouch young of age 0, 10 and 15 days *post partum*. As the structure of the VNO and MOE are not visible under the dissecting microscope at the age of 0 to 15 days, the areas in which they lie were dissected out. All samples were snap frozen with liquid nitrogen and stored at -80°C until use.

### RNA extraction and RT-PCR

RNA was extracted using the RNeasy Lipid Tissue Mini kit (QIAGEN, Doncaster Victoria, Australia; #74804) for VNO and brain tissue and TRI-reagent for the other tissues. RNA was DNase-treated (Ambion, USA; #AM1906). For each sample, 8 μg was reverse transcribed using SuperScript™III (Invitrogen; Mount Waverley, Victoria, Australia; #12574-018) in a total reaction volume of 20 μL.

For cloning and sequencing of cDNA fragments, an initial PCR was performed using primers (1F and 1R) spanning Exons 2-23 (Table 1). PCR was performed using ExTaq Polymerase (TaKaRa) according to the manufacturer's instructions, in a 20 μL volume containing 0.5 μL of 1st strand cDNA template as follows: 95°C for 1 min.; 40 cycles of 95°C for 20 sec, 60°C for 20 sec., 68°C for 4 min.; 68°C for 1 min. Nested PCRs were performed using identical conditions to above except that different primers were used (Table 1) and the template was 0.5 μL of the initial PCR. RT-PCR products were cloned to pGEM-T-Easy (Promega, NSW, Australia) for sequencing.

For tammar expression studies, PCR was performed using GoTaq Green (Promega, NSW, Australia) according to the manufacturer's instructions, in a 10-μL volume containing 0.5 μL of 1st strand cDNA template as follows: 95°C for 1 min.; 35 cycles of 95°C for 20 sec, 60°C for 20 sec., 72°C for 1 min.; 72°C for 1 min. For platypus expression studies, PCR conditions were identical except that ExTaq Polymerase was used according to the manufacturer's instructions and the annealing temperature was 68°C. Primer sequences were as indicated in Tables 1 and 2.

**Table 2:** Sequences of platypus *XNDR-TRPC2*-specific primers

Gene	name	sequence	location of primer binding
*XNDR*	p1F	GCAGAGGACGGAGAGATGGGTCAAG	Exon 9
	p1R	CGAGGAGGGGCAGGAGTGGAAAC	Exon β

*TRPC2*	p2F	GGAGACGGCACAGGGAACGG	Exon 10
	p2R	TTGAGCACGGCGGTCACCTC	Exon 13

### Sequence analysis

NCBI BLAST http://blast.ncbi.nlm.nih.gov/Blast.cgi was used to search whole genome shotgun (WGS) databases and trace archives. The UCSC browser http://www.genome.ucsc.edu/ was used to search and analyse assembled genomes. Contigs were assembled from trace archive sequences using CAP3 http://pbil.univ-lyon1.fr/cap3.php. Identification of exons was aided by use of GENSCAN http://genes.mit.edu/GENSCAN.html. Sequence alignments were performed using ClustalW in the MacVector software package. Tammar sequences assembled from cloned cDNA fragments were deposited in GenBank as follows: *TRPC2 *[GenBank:GQ860951], *XNDR *isoform 1 [GenBank:GQ860953], *XNDR *isoform 2 [GenBank:GQ860953]. Sequences for promoter analysis were taken from genome builds on the UCSC browser for all species, except tammar wallaby, as follows: mouse build *mm9*, chr7:109231513-109231712; human build *hg19*, chr11:3636855-3636875; dog build *canFam2*, chr21:29188689-29188900; cow build *bosTau4*, chr15:51030125-51029929; elephant build *loxAfr3*, scaffold_79:7861327-7861525; opossum build *monDom5*, chr4:348474319-348474426; platypus build *ornAna1*, Contig17150:12022-12235; and tammar wallaby [GenBank:ABQO011015996], 388-206.

## Authors' contributions

The experiments were conducted and evaluated by SF and NYS. NYS, MBR, TF and GS assisted in the collection of tissues. SF, NYS and MBR prepared the manuscript. All authors discussed the results and commented on the manuscript. All authors read and approved the final manuscript.
